# Effectiveness of Combined Preoperative and Postoperative Rehabilitation Versus Postoperative-Only Rehabilitation in Cardiac Surgery Patients: A Systematic Review

**DOI:** 10.7759/cureus.98060

**Published:** 2025-11-29

**Authors:** Sherif Elbadrawy, Muneeba Gul, Hesham Abdelwahed

**Affiliations:** 1 Critical Care Medicine, Wellington Regional Hospital, Wellington, NZL; 2 Rehabilitation, Rehman Medical Institute, Peshawar, PAK; 3 Critical Care, Maitland Hospital, Newcastle, AUS; 4 Critical Care Medicine, Newcastle University, Newcastle, AUS

**Keywords:** cardiac surgery, functional capacity, postoperative complications, postoperative rehabilitation, preoperative rehabilitation, pulmonary function, systematic review

## Abstract

Prehabilitation, delivered before surgery in addition to standard postoperative rehabilitation, has been proposed as a strategy to improve outcomes in patients undergoing cardiac surgery. While postoperative rehabilitation is standard of care, the incremental benefit of adding a preoperative phase remains uncertain.

We conducted a systematic review and meta-analysis of randomized controlled trials (RCTs) and observational studies comparing combined preoperative and postoperative rehabilitation (Pre+Post) with postoperative rehabilitation alone (Post-only) in adults undergoing elective cardiac surgery. Comprehensive searches of PubMed, CINAHL, Cochrane, and MEDLINE were performed up to March 2025. Primary outcomes were postoperative pulmonary complications (PPCs), functional capacity, pulmonary function, and length of hospital stay (LOS). Secondary outcomes included ICU stay, duration of mechanical ventilation, quality of life (QoL), and psychological measures. The risk of bias was assessed using the RoB 2 tool for RCTs. Pooled analyses were conducted using random-effects models.

Seventeen studies met the inclusion criteria, comprising 11 RCTs and six non-randomized studies, with over 3,200 participants. Meta-analysis of six RCTs (n = 1,729) showed that Pre+Post rehabilitation significantly reduced PPCs (RR 0.39, 95% CI 0.19-0.80, p = 0.01), corresponding to a 61% lower risk. Four RCTs (n = 315) demonstrated superior postoperative functional capacity in the Pre+Post group, with a pooled mean difference of +42 m in 6MWT (95% CI 32.39-51.68, p < 0.00001, I² = 0%). Pulmonary function was improved, with increases in FVC (+0.45 L, 95% CI 0.20-0.70) and FEV₁ (+0.36 L, 95% CI 0.14-0.58). Hospital LOS was reduced by almost three days (MD -2.92, 95% CI -4.52 to -1.31), though heterogeneity was high (I² = 87%). Effects on ICU stay and QoL were mixed.

Adding a structured prehabilitation phase to standard postoperative rehabilitation significantly reduces PPCs, improves functional capacity, and enhances pulmonary function after cardiac surgery. While benefits on LOS and QoL are less consistent, the overall evidence supports the integration of prehabilitation into perioperative care pathways. Future large-scale RCTs with standardized protocols are warranted.

## Introduction and background

Cardiac surgery is considered a critical intervention for the management of intricate cardiovascular disorders such as valvular disorders and coronary artery disease. Despite that, postoperative complications affecting multiple organ systems, like gastrointestinal, respiratory, and neurological functions, remain a high risk for patients undergoing cardiac surgery [1]. These complications can markedly prolong recovery, hospital and ICU stays, increase morbidity, and healthcare expenses.

In consequence of these complications, cardiac rehabilitation (CR) has evolved into a multidisciplinary approach aimed at improving surgical outcomes through techniques such as physical therapy, patient education, nutritional guidance, respiratory exercises, and psychosocial support. It has been observed that CR increases exercise tolerance, enhances functional status, decreases mortality, and postoperative disability [2]. Early mobilization, reduced stay in ICU, and collaboration of a comprehensive team are considered as guiding principles of effective rehabilitation [3].

The use of preoperative rehabilitation, also termed prehabilitation, is now supported by an increasing amount of research. It basically includes physical as well as respiratory conditioning prior to surgery. This intervention has been shown to reduce the risk of postoperative respiratory complications, limit hospital stays, and improve functional capacity and pulmonary function [4,5]. This approach of prehabilitation is specifically helpful for older individuals and patients at increased risk of respiratory complications. Though its effects on atrial fibrillation and perioperative mortality are still unclear, its positive effects in improving recovery are recognizable [6].

Moreover, postoperative cardiac rehabilitation has shown efficacy in improving functional performance and recovery. One study mentioned increased functional capacity in patients who took part in postoperative CR programs [7]. Another study has highlighted that early, comprehensive inpatient CR programs result in marked improvements in functional status for patients with severe disability, as well as a 50% reduction in five-year mortality [8]. However, some studies have cited mixed results regarding the effect of CR’s on mortality [9]. Studies generally support improvement in quality of life (QoL) and modest reductions in hospital stay [6].

Programs using a combination of preoperative and postoperative rehabilitation may provide additional advantages. One paper states that preoperative respiratory rehabilitation combined with postoperative care results in improved pulmonary outcomes, reduced duration of mechanical ventilation, and unregulated overall recovery [10]. In 2017, Gomes Neto also highlighted that the combined use of preoperative and postoperative inspiratory muscle training (IMT) reduces complications and improves pulmonary function [11]. However, the credibility of this evidence differs, as some show moderate to high risk of bias or heterogeneity in intervention protocols.

The implementation of cardiac rehabilitation, despite having so many benefits, is still underutilized and inconsistent. Mampuya cited minimal inclusion of participants due to inadequate referral systems and limited awareness of patients [12]. Other authors highlighted constraints at the provider level (e.g., poor incorporation into discharge planning), at the patient’s end (e.g., psychosocial issues, comorbidities, travel limitations), and at the health system level (e.g., limited well-equipped staff, limited resources) [13,14]. In settings having limited resources, such as India, Babu cited multiple obstacles, including limited professional awareness and a lack of support by institutions for CR [15]. To limit these obstacles, researchers recommend improvement of referral processes, dedicated allocation of resources, a multidisciplinary team approach, and individualized treatment programs [16,17].

In light of these potential advantages of combined preoperative and postoperative rehabilitation in cardiac surgery patients, it is necessary to systematically identify whether a combined approach yields better results in contrast to postoperative rehabilitation only. This systematic review aims to fill this gap by assessing the implications of combined preoperative and postoperative rehabilitation versus postoperative-only programs on pulmonary function, early mobilization, length of hospital/ICU stay, and related recovery outcomes in adult cardiac surgery patients.

## Review

Methods

Eligibility Criteria

This systematic review included studies involving adult patients (aged 18 years and older) undergoing elective cardiac surgery, such as coronary artery bypass grafting (CABG), valve replacement, or combined procedures. Eligible studies examined the effect of preoperative rehabilitation, postoperative rehabilitation, or a combination of both. Interventions included structured physical therapy, IMT, aerobic exercise programs, or other physiotherapy-based rehabilitation protocols. Studies were eligible if they compared these interventions to usual care, postoperative rehabilitation alone, or no rehabilitation and reported at least one relevant outcome, such as pulmonary function, functional mobility, QoL, or postoperative recovery indicators.

We included randomized controlled trials (RCTs) published in English from January 2000 to March 2025. Studies were excluded if they involved non-cardiac surgeries, pediatric populations, animal subjects, or were presented as case reports, editorials, conference abstracts, or review articles (such as systematic reviews or meta-analyses). Studies that lacked full-text availability or did not report relevant outcome data were also excluded.

Information Sources

A comprehensive literature search was conducted across four electronic databases: PubMed, MEDLINE, CINAHL, and the Cochrane Library (CENTRAL). The searches were performed between March 20 and March 22, 2025. Additional sources such as clinical trial registries, websites, or reference lists were not used in this review.

Search Strategy

We developed detailed search strategies for each database using a combination of free-text terms and controlled vocabulary where available. Search terms focused on key concepts related to cardiac surgery, preoperative and postoperative rehabilitation, and RCTs. An example search string used in PubMed included terms such as “cardiac surgery,” “coronary artery bypass,” “prehabilitation,” “postoperative rehabilitation,” and “randomized controlled trial.” The full Search string was : ("cardiac surgery" OR "heart surgery" OR "CABG" OR "valve replacement") AND ("preoperative rehabilitation" OR "postoperative rehabilitation") AND ("functional recovery" OR "ICU stay" OR "length of stay" OR “pulmonary function” OR “early mobilization”). Search strategies were adapted as appropriate for each database. Filters were applied to limit results to human studies published in English from 2000 onward.

Selection Process

All retrieved records were exported into a reference management tool, and duplicates were identified and removed manually. Two reviewers independently screened the abstracts; one reviewer (SE) screened the titles and abstracts of all unique records to identify eligible studies. Full-text articles were retrieved for studies that appeared to meet the inclusion criteria or lacked sufficient information in the abstract. These full texts were assessed in detail to confirm eligibility. A second senior reviewer (HA) screened the studies against the inclusion criteria to eliminate bias.

Data Collection Process

Data extraction was performed independently by two reviewers using a predesigned structured data extraction form. The form captured key study characteristics, including study design, setting, population, intervention details, comparison group, reported measured outcomes, and main results. Extracted data were cross-checked against the full-text articles to ensure accuracy and completeness. No automation tools were used, and the study authors were not contacted for additional information.

Data Items

For each included study, we extracted outcome data relevant to the objectives of this review. Outcomes included pulmonary function tests such as forced vital capacity (FVC), forced expiratory volume in one second (FEV₁), and peak expiratory flow (PEF), functional mobility measures such as the six-minute walk test (6MWT) and Timed-Up-and-Go (TUG) test, postoperative complications including pneumonia and reintubation, ICU and hospital length of stay, and QoL scores. When multiple time points were reported, we focused on the latest available postoperative values.

In addition to outcomes, we collected information on sample size, participant demographics, type and duration of rehabilitation interventions, trial registration status, and reported funding sources. When studies presented incomplete or ambiguous data, assumptions were clearly noted.

Risk of Bias Assessment

The risk of bias of included studies was assessed according to study design. For RCTs, we used the Cochrane Risk of Bias 2 (RoB 2) tool, which evaluates bias across five domains: randomization process, deviations from intended interventions, missing outcome data, measurement of the outcome, and selection of the reported result. For non-randomized studies (retrospective or quasi-experimental designs), we used the ROBINS-I tool, which assesses risk of bias in seven domains: confounding, selection of participants, classification of interventions, deviations from intended interventions, missing data, measurement of outcomes, and selection of reported results. Judgments were made at the domain level and summarized into an overall risk-of-bias rating. Visual traffic-light plots were generated using the robvis tool.

Results

Study Selection

A total of 427 records were identified through database searches (PubMed = 246, CINAHL = 76, Cochrane = 77, Medline = 28). After removing 21 duplicates, 406 records remained for title and abstract screening, resulting in the exclusion of 252 studies. Of the 154 full-text articles sought for retrieval, 72 were not retrieved due to being conference abstracts, involving non-cardiac surgeries, or trial registrations at the time of search.

**Figure 1:**
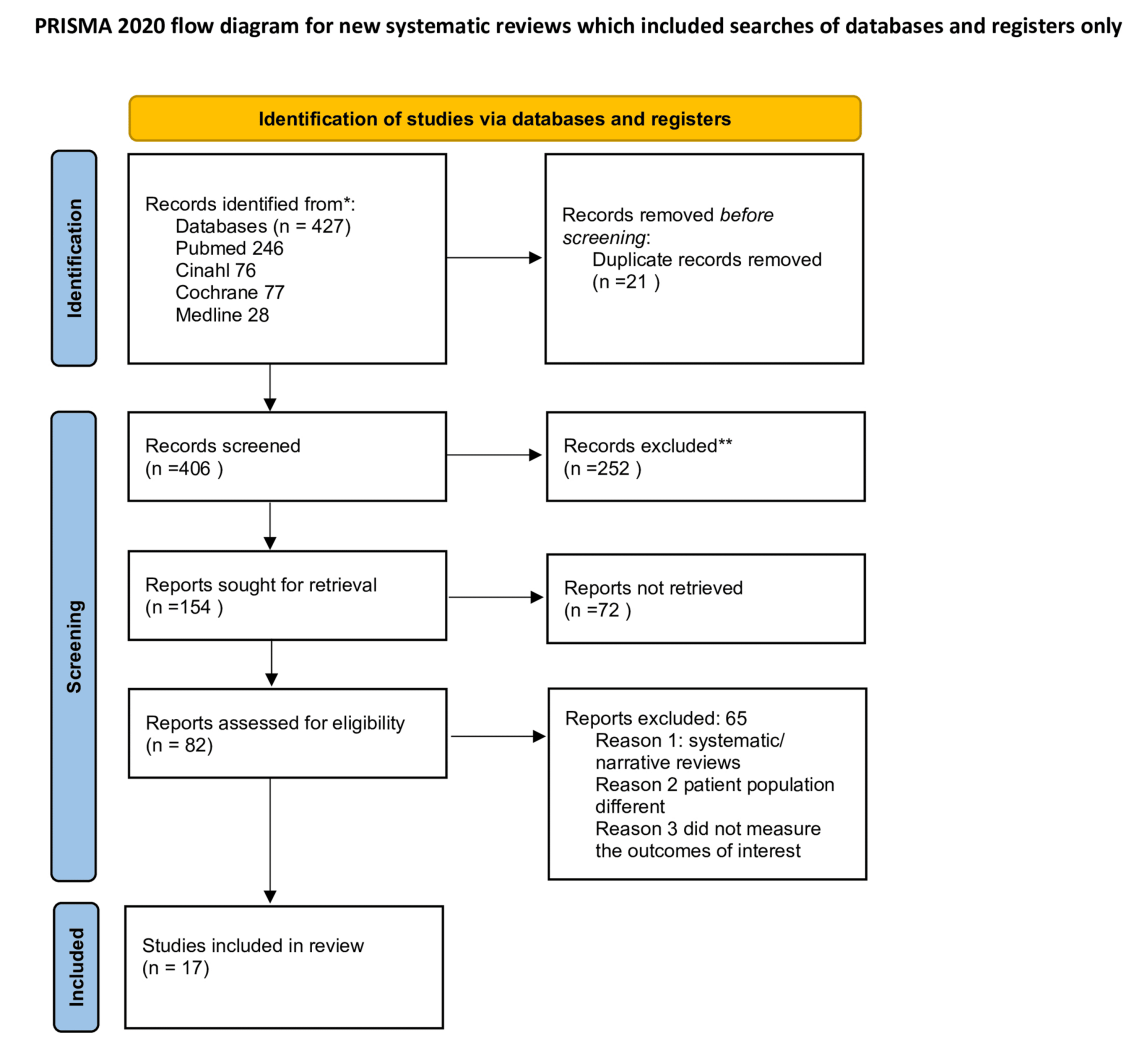
Preferred Reporting Items for Systematic Reviews and Meta-Analyses (PRISMA) Flow Diagram. *Consider, if feasible to do so, reporting the number of records identified from each database or register searched (rather than the total number across all databases/registers). **If automation tools were used, indicate how many records were excluded by a human and how many were excluded by automation tools.

The remaining 82 full-text articles were assessed for eligibility; of these, 65 were excluded for reasons such as being narrative or systematic reviews, lack of full-text availability, or having a restricted patient population (e.g., only male or only female). Ultimately, 17 studies met the inclusion criteria and were included in the final synthesis. The study selection process is illustrated in Figure 1.

A total of seventeen studies, comprising eleven RCTs and six non-randomized studies, were included in this review. Collectively, these studies enrolled more than 3,200 patients undergoing cardiac surgery, predominantly CABG with or without concomitant valve procedures. The trials were geographically diverse, spanning Europe, Asia, and North America, and evaluated a broad range of prehabilitation strategies, including IMT, multimodal exercise and education programs, neuromuscular electrical stimulation (NMES), nutritional optimisation, and psychological counselling. In all RCTs, the intervention compared combined preoperative and postoperative rehabilitation (Pre+Post) with postoperative rehabilitation alone (Post-only), reflecting the clinical reality that postoperative rehabilitation is routinely provided to all patients (Table 1).

**Table 1:** Characteristics of Included Studies.

First Author (Year)	Country	Surgery Type	Sample Size (IG/CG)	Design	Intervention Details	Comparator Details	Duration/Frequency	Notes
Hulzebos et al. (2006) [5]	Netherlands	CABG (high-risk)	139/137	RCT	IMT: 20 min/day, 6 days/week, ≥2 weeks; + incentive spirometry, breathing	Usual care: preop breathing/coughing education	2-10 weeks preop	JAMA, registered. Outcomes: PPCs, pneumonia, LOS
Nejkov et al. (2020) [10]	Montenegro	CABG/valve	11/8	RCT (small sample)	Pre + postop rehab: diaphragmatic breathing, postural ed., counseling	Postop rehab only	~7 days preop	Tiny sample. Outcomes: LOS, MV duration, functional tests
Khushnood et al. (2023) [18]	Pakistan	CABG	25/25	RCT	Preop physiotherapy: IMT + breathing + chest clearance; 3 sessions × 30-40 min	Breathing + chest clearance only	3 preop sessions	Outcomes: 6MWT, kinesiophobia, ICU stay
Niazi et al. (2022) [19]	Iran	CABG/valve	28/26	RCT (low quality)	Preop pulmonary rehab (IMT, IS, breathing, mobilization) + psychological counseling	Usual preop drug treatment only	≥10 days preop	Outcomes: ICU/hospital stay, stress, depression
Shakouri et al. (2015) [20]	Iran	CABG	30/30	RCT	15 sessions: breathing, IS, thoracic mobility, muscle tension/shoulder ex.	Postop physiotherapy only	2 weeks preop	Registered. Outcomes: FVC, FEV1, ABG, ICU stay, MV duration
Gnanavelu et al. (2024) [21]	India	CABG/valve	50/50	RCT	Prehab: aerobic/resistance ex. (3×/week), nutrition, psych support	Standard care	4 weeks preop	Outcomes: ↓ complications, LOS, better 6MWT and Karnofsky
Savci et al. (2011) [22]	Turkey	CABG	22/21	RCT	IMT with Threshold device, 2×/day, 30 min; 5 days preop + 5 days postop	Usual physiotherapy (mobilization, breathing, coughing, ambulation)	10 days	Outcomes: MIP, 6MWT, ICU stay, anxiety, QoL
Scheenstra et al. (2025) [23]	Netherlands (multicenter)	CABG, valve, aortic, TAVR	197/197	RCT	Tele-prehab: exercise, IMT, nutrition, psych, smoking cessation	Standard care	6-12 weeks preop	Multicenter JACC. Outcomes: ↓MACE at 1y, QoL, LOS/complications (ns)
Sumin et al. (2023) [24]	Russia	CABG/valve	62/60	RCT	NMES of quadriceps (Beurer EM80), 7-10 daily sessions × 90 min	Standard care: breathing + education	7-10 days preop	Registered. Outcomes: ↑ muscle strength, 6MWT
Arthur et al. (2000) [25]	Canada	CABG (low-risk)	123/123	RCT	Exercise 2×/week, 90 min; education; nurse calls; smoking cessation	Usual waitlist care	~8 weeks preop	Ann Intern Med. Outcomes: ↓ LOS, ↓ ICU time, ↑ QoL
Valkenet et al. (2016) [26]	Netherlands	CABG (high-risk)	119/116	RCT	Home IMT: 20 min/day, 7 days/week; weekly supervised; 30% Pimax	Breathing/mobilization education only	2-4 weeks preop	ISRCTN. Outcomes: ↓ pneumonia, ↓ LOS, QoL (no added effect)
Montero-Cámara et al. (2025) [27]	Spain	CABG/valve	418	Retrospective cohort	Single 2-hr prehab session + home program	Usual care	~3 weeks preop	↓ LOS, ↓ mortality risk
Umar et al. (2024) [28]	Pakistan	Mitral valve	40	Quasi-experimental	RMT for 3 weeks preop	Conventional breathing exercises	3 weeks preop	Improved QoL, shorter MV duration
Waite et al. (2017) [29]	UK	CABG/valve (frail)	22	Single-group pilot	Home-based PREHAB (Otago-based balance and strength ex.)	None	~6 weeks preop	Improved 6MWT, frailty score, ↓ LOS
Nardi et al. (2020) [30]	Italy	CABG/valve/aortic	102 (34/34/34)	Retrospective controlled	Respiratory (R) vs Respiratory+Motor (R+M) physiotherapy protocols	Simplified physiotherapy (basic breathing + assisted walking)	Pre + postop	↓ LOS, ↑ 6MWT, improved blood gases
Nakamura et al. (2024) [31]	Japan	CABG post-ACS	393 (26 vs 26 matched)	Retrospective matched cohort	In-hospital rehab: mobilization, aerobic, resistance, stretching, nutrition, psych support	Standard care	23 ± 12 days preop (ACS admission)	Safe, feasible; no significant LOS or outcomes difference
Sobczyk et al. (2024) [32]	Poland	CABG/valve	725 (338/387)	Prospective observational	Pre S check: outpatient multidisciplinary + home program (respiratory + mobility ex. 20 min/day, 2-3 weeks)	Standard care	2-3 weeks preop	↓ Pneumonia, ↓ resp. failure, ↓ complications

**Figure 2:**
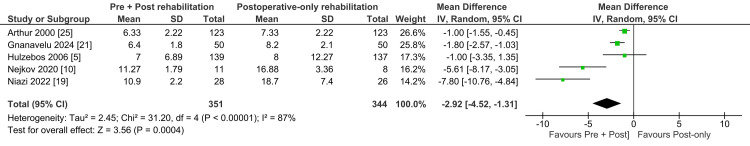
Effect of Combined Preoperative and Postoperative Rehabilitation on Length of Hospital Stay in Cardiac Surgery Patients.

Length of hospital stay (LOS) was among the most frequently reported outcomes. Multiple well-conducted RCTs demonstrated that the addition of a prehabilitation phase shortened total hospitalization compared with postoperative-only rehabilitation. Two studies reported approximately two-day reductions in LOS among intervention groups [5,26]. Similarly, another study showed that Canadian patients randomized to a supervised preoperative exercise and education program experienced shorter postoperative hospitalizations and were discharged earlier than controls [25]. Similar findings were reported by another study from Pakistan that preoperative rehabilitation in MVR patients led to reduced LOS [28]. Consistent findings were observed in smaller RCTs from Iran [19,20] and Montenegro [10], all of which favored the addition of prehabilitation. Observational studies provided further support, reporting reduced LOS among patients who engaged in structured preoperative programs (Figure 2) [27,29,30,32].

This meta-analysis of five RCTs (n = 695) demonstrated that patients receiving combined preoperative and postoperative rehabilitation had significantly shorter hospital stays compared with those receiving postoperative rehabilitation alone. The pooled mean difference was -2.92 days (95% CI: -4.52 to -1.31; p = 0.0004), favoring the combined intervention. Although heterogeneity was high (I² = 87%), the direction of effect was consistent across studies, with all included trials showing reductions in length of stay in the Pre+Post group. Notably, the greatest benefit was observed in smaller studies from Iran and Montenegro [10,19], whereas larger European trials [5,25] showed more modest reductions.

**Figure 3:**
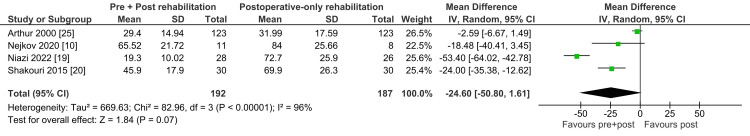
Effect of Combined Preoperative and Postoperative Rehabilitation Versus Postoperative-Only Rehabilitation on ICU LOS and Duration of Mechanical Ventilation.

ICU length of stay and duration of mechanical ventilation were also improved in the combined Pre+Post rehabilitation groups. One study demonstrated significantly shorter ICU stays among patients who participated in supervised preoperative exercise [25]. Similarly, another author reported both reduced ICU stay and shorter mechanical ventilation duration with the addition of prehabilitation [20], while others observed similar benefits in Turkey using a combined preoperative and postoperative IMT protocol [22]. Smaller RCTs likewise showed earlier extubation and faster ICU recovery in their intervention groups [10,19]. Observational evidence was more heterogeneous: for example, in Japan, using a propensity-matched design, they found no significant differences in ICU outcomes, although their findings confirmed the safety and feasibility of implementing prehabilitation even in patients admitted with acute coronary syndrome (Figure 3) [31].

Four RCTs comprising 379 patients (192 intervention, 187 control) reported on ICU length of stay. The pooled analysis using a random-effects model showed no statistically significant difference between combined preoperative and postoperative rehabilitation and postoperative-only rehabilitation (mean difference -24.60 hours, 95% CI -50.80 to 1.61; p = 0.07). While the direction of effect favored combined rehabilitation, the confidence interval (CI) crossed the line of no effect, precluding firm conclusions. Heterogeneity was considerable (I² = 96%), likely reflecting differences in ICU practices, patient characteristics, and rehabilitation protocols across studies.

**Figure 4:**
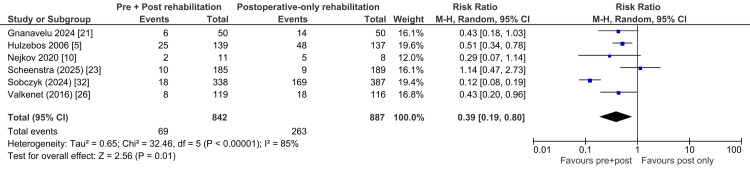
Effect of Combined Preoperative and Postoperative Rehabilitation Versus Postoperative-Only Rehabilitation on Postoperative Complications.

Postoperative pulmonary complications (PPCs) were a central outcome across multiple trials. For example, a landmark JAMA study demonstrated that high-risk CABG patients who underwent preoperative IMT had a significantly lower incidence of PPCs, including pneumonia, compared with controls [5]. Similarly, one author in India found that prehabilitation was associated with lower overall complication rates [21]. Observational data lend additional support: In a large Polish cohort, another author reported marked reductions in respiratory failure and pneumonia following the establishment of a multidisciplinary preoperative clinic (Figure 4) [32].

PPCs were reported in six trials, encompassing a total of 1,729 patients (842 in the intervention group and 887 in the control group). Pooled analysis demonstrated a significant reduction in PPCs with combined preoperative and postoperative rehabilitation compared with postoperative-only rehabilitation (risk ratio (RR) 0.39, 95% CI 0.19-0.80; p = 0.01), corresponding to a 61% lower risk in the intervention group. Heterogeneity was substantial (I² = 85%), likely reflecting variations in study size, intervention protocols, and patient risk profiles. Despite this variability, the overall direction of effect consistently favored combined rehabilitation strategies.

**Figure 5:**
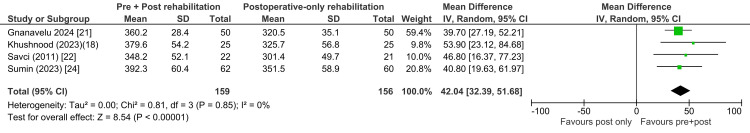
Effect of Combined Preoperative and Postoperative Rehabilitation Versus Postoperative-Only Rehabilitation on Functional Capacity Assessed by 6MWT.

Functional capacity was consistently enhanced with the addition of prehabilitation. One study demonstrated substantial increases in 6MWT and inspiratory muscle strength in patients who underwent IMT before and after surgery [22]. Similar improvements were reported by other studies with intervention groups achieving better postoperative 6MWT performance and muscle strength [18,24]. One study from India also observed higher 6MWT distances and improved Karnofsky Performance Status at 30 days [21], while one study reported significant gains in physical functioning measured by the SF-36, alongside greater participation in formal cardiac rehabilitation programs after discharge [25]. A non-randomized study in frail patients also demonstrated meaningful improvements in mobility and endurance (Figure 5) [29].

Functional capacity, assessed using the 6MWT, was evaluated in four RCTs involving 315 patients (159 in the intervention group and 156 in the control group). Pooled analysis demonstrated a significant improvement in walking distance for patients receiving combined preoperative and postoperative rehabilitation compared with postoperative-only rehabilitation (mean difference 42.04 m, 95% CI 32.39-51.68; p < 0.00001). No heterogeneity was observed across trials (I² = 0%), indicating highly consistent results. These findings provide robust evidence that combined rehabilitation significantly enhances postoperative functional recovery.

**Figure 6:**
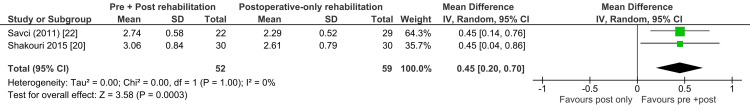
Effect of Combined Preoperative and Postoperative Rehabilitation Versus Postoperative-Only Rehabilitation on Pulmonary Function Tests Assessed by FVC.

**Figure 7:**
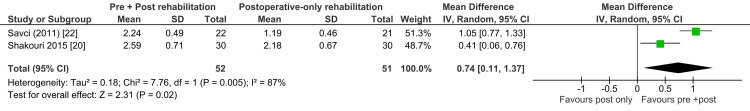
Effect of Combined Preoperative and Postoperative Rehabilitation Versus Postoperative-Only Rehabilitation on Pulmonary Function Tests Assessed by FEV1.

Pulmonary function and respiratory muscle performance outcomes further supported the benefits of combined rehabilitation. One study reported that prehabilitation resulted in higher postoperative FVC, forced expiratory volume in 1 second (FEV₁), and PEF, alongside improved arterial oxygenation [20]. Savci et al. observed significant increases in maximal inspiratory pressure [22], while another study demonstrated enhanced respiratory muscle endurance and a reduction in postoperative PPCs, highlighting the clinical significance of these physiological improvements (Figure 6 and Figure 7) [5,22].

Pulmonary function was assessed in two RCTs using spirometric indices. Pooled analysis of 111 patients for FVC demonstrated that combined preoperative and postoperative rehabilitation significantly improved postoperative FVC compared with postoperative-only rehabilitation (mean difference 0.45 L, 95% CI 0.20-0.70; p = 0.0003; I² = 0%). Similarly, pooled analysis of 103 patients for FEV₁ showed significantly higher postoperative FEV₁ in the intervention group (mean difference 0.36 L, 95% CI 0.14-0.58; p = 0.001; I² = 0%). No heterogeneity was observed in either analysis, indicating highly consistent results. These findings indicate that combined rehabilitation effectively enhances postoperative pulmonary function, as reflected by improvements in both FVC and FEV₁.

QoL and psychological outcomes were assessed in several studies. One study reported sustained improvements in health-related QoL at six months in the intervention group [25]. Another author observed enhanced sleep quality and reduced anxiety scores [22]. More recent studies demonstrated that using a tele-prehabilitation model resulted in better one-year QoL scores, lower depression rates, and improved risk factor control, including smoking cessation, among patients who participated in prehabilitation [23]. Similarly, other authors reported improvements in functional recovery and patient-reported outcomes with multimodal prehabilitation [21]. In contrast, one study found that although QoL improved in both groups postoperatively, prehabilitation did not confer additional benefit beyond natural recovery [26]. In psychological domains, one study documented lower preoperative stress and depression in patients who received counselling combined with preoperative respiratory training [19].

Risk of Bias Assessment

A total of 17 studies were included in the review: 11 RCTs assessed with RoB 2 and six non-RCTs assessed with ROBINS-I.

Among the 11 RCTs, most were judged to have some concerns of bias, particularly in domains related to the randomization process and selective reporting. One trial [10] was judged at high risk of bias due to unclear sequence generation and allocation concealment, while one large multicenter RCT [23] was judged at low risk of bias across all domains.

**Figure 8:**
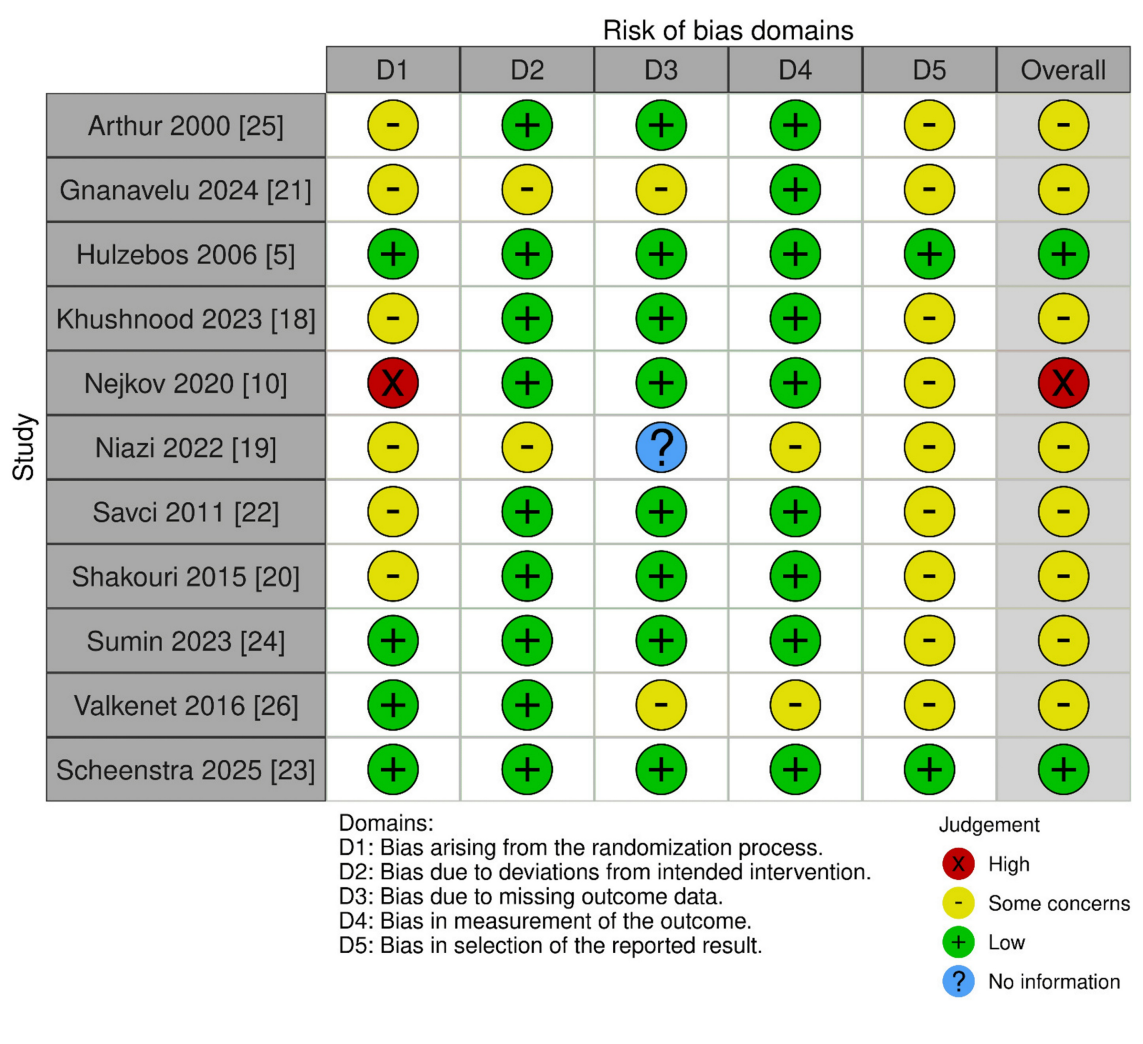
Risk of Bias Assessment RCT.

**Figure 9:**
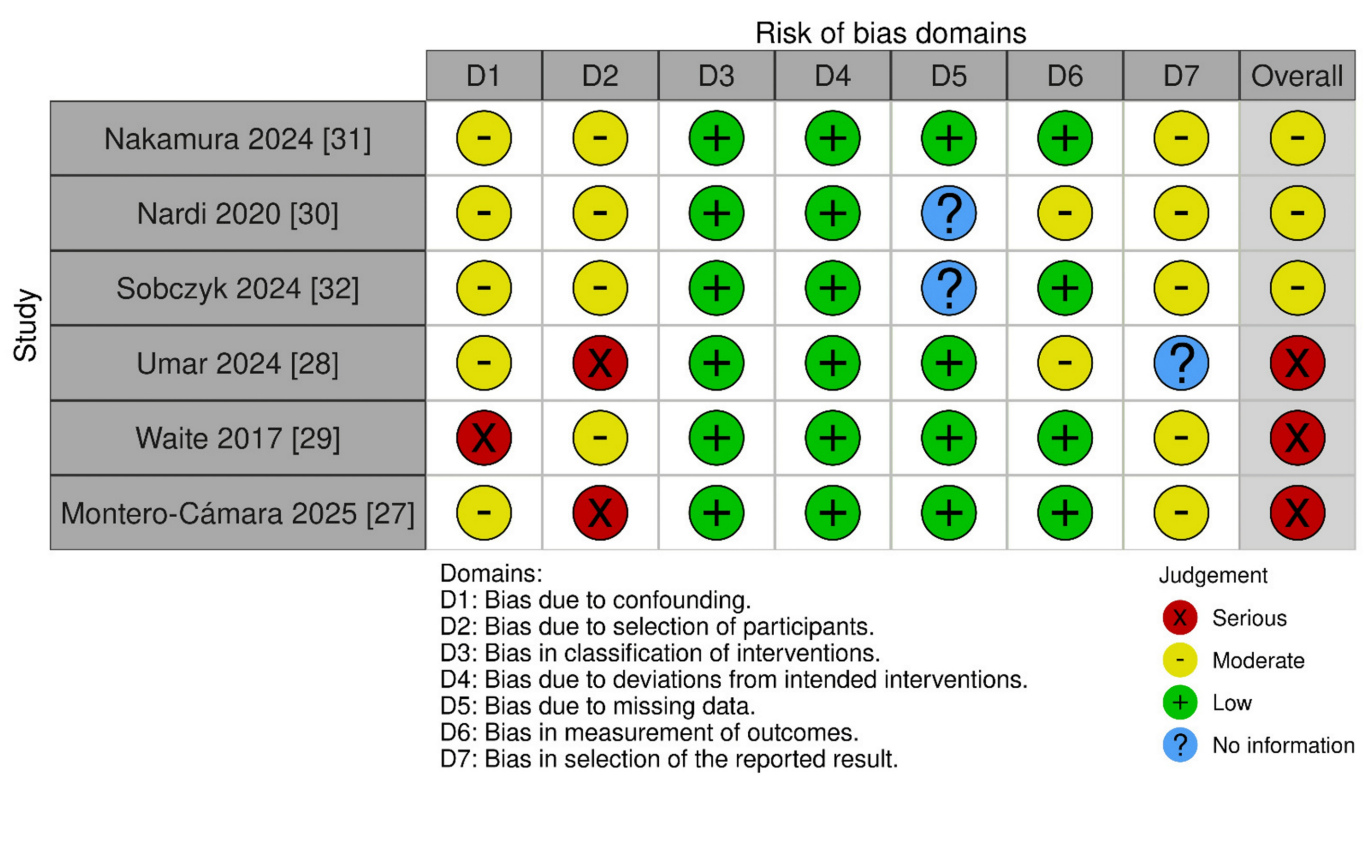
Risk of Bias Assessment Non-RCT.

Among the six non-RCTs, three RCTs had a moderate risk of bias, and three had a high risk of bias, mainly due to bias in the first (confounding) and second(participant selection) domains. These assessments are summarized in Figure 8 and Figure 9.

Discussion

This systematic review and meta-analysis reveal that the combination of standard postoperative rehabilitation with prehabilitation results in clinically significant benefits for cardiac surgery patients. Consistent association of prehabilitation with reduction in postoperative respiratory complications, improvement in pulmonary function indices like FEV₁ and FVC, and enhanced functional recovery as determined by the 6MWT is highlighted by multiple randomized controlled trials. Even though the influence on the duration of hospital and ICU stays was not uniform, overall, the benefits of using a combined approach were favored.

Our findings support the influential contribution of previous authors, who first cited the reduction in postoperative PPCs after using preoperative IMT in a high-risk CABG population [5]. These outcomes are supported by the present pooled analysis that shows a 61% relative reduction of risk in PPCs among six randomized control trials. Similar outcomes are reported by Durkin [26], particularly highlighting a decrease in pneumonia, which coincides with our effect estimates in the face of the moderate heterogeneity. In some more recent RCTs, such as Gnanavelu et al [21], reductions are observed, reflecting the reproducible nature of these benefits in multiple healthcare settings and current standards of surgical care

Functional capacity outcomes reflected substantial effects, demonstrating, on average, an increase of around 40 m on the 6MWT in the intervention group. This is both clinically and statistically important, exceeding the minimal clinically important difference (MCID) often published for pulmonary and cardiac rehabilitation populations (20-30 m). The reliability of this evidence is emphasized by the lack of heterogeneity across included trials. Specifically, these findings are in accordance with studies from other surgical populations: a 2023 meta-analysis by Jansen et al. in patients undergoing major abdominal surgery also highlighted improvements related to prehabilitation in functional recovery at discharge, indicating a generalizable physiological benefit of preoperative optimization of patients.

Pulmonary function indices further supported these advantages. Increases of 0.45 L in FVC and 0.36 L in FEV₁, observed in our findings, are clinically relevant and suggestive of enhanced postoperative respiratory mechanics. These results are in accordance with prior physiological studies indicating that IMT improves respiratory endurance and decreases the likelihood of atelectasis and pneumonia. Besides randomized control trials, observational cohorts such as Sobczyk et al. [32] demonstrated the likely real-world effect, highlighting a significant decrease in respiratory failure after imposition of a structured prehabilitation clinic.

Finding for decreased length of hospital/ICU stay was less conclusive. Though individual RCTs such as Hulzebos et al. [5] and Arthur et al. [25] cited significant reductions, the pooled analyses showed high heterogeneity, with CIs crossing the line of no effect in ICU outcomes. These inconsistencies can be explained by several factors, such as differences in the complexity of surgeries, perioperative practices, policies of discharge, and definitions of ICU stay across healthcare systems. Large-scale registry-based analyses, such as Nakamura et al. [31] in Japan, have also highlighted impartial effects on ICU metrics, emphasizing that system-level variables may reduce the quantifiable effects of prehabilitation on these results. However, the persistent direction of advantage observed across most studies indicates that prehabilitation likely helps in earlier mobilization and recovery, even if these benefits are not completely captured in standardized metrics like length of stay in ICU, QoL outcomes remain undetermined. While Arthur et al. [25] and Savci et al. [22] figured out constant improvements in QoL and psychological health, Valkenet et al. [26] cited no substantial advantage other than trajectories in natural recovery. Still, growing evidence from current trials using tele-prehabilitation models [23] revealed that constant improvements in QoL and mental well-being may be achievable when treatments are implemented into the home environment. This shows an emerging model where hybrid prehabilitation models, incorporating physical, nutritional, and psychological elements, may result in the most long-lasting benefits.

In a nutshell, this review illustrates that prehabilitation may confer enhancements in clinical outcomes most directly related to physiological reserve, namely, PPCs, functional capacity, and spirometry. The benefits of QoL and length of stay, while encouraging, remain dependent on context and are impacted by system-related factors. Our results indicate that incorporating structured prehabilitation into perioperative care pathways has the potential to improve recovery possibilities, specifically when multimodal and adapted for patient risk profiles.

Limitations

This review has several limitations. First, some potentially relevant studies could not be retrieved because they were published only as conference abstracts, involved non-cardiac surgical populations, or were registered trials without available full-text results. Second, the review process was conducted by a single reviewer, which may have introduced selection bias, although prespecified eligibility criteria were strictly applied. Third, the number of RCTs available for certain outcomes, particularly spirometric indices and QoL, was limited, which reduces the precision and generalizability of pooled estimates. Fourth, although non-randomized studies were excluded from meta-analyses, their inclusion in the qualitative synthesis may have introduced some degree of bias. Fifth, this review was limited to studies published in English, which may have excluded relevant evidence from other languages. Additionally, some reported outcomes were surrogate measures (e.g., spirometric parameters or short-term functional tests) rather than long-term, patient-centered outcomes, which may restrict the interpretation of clinical significance. Finally, the possibility of publication bias cannot be excluded, as smaller negative trials may remain unpublished.

## Conclusions

In summary, this systematic review and meta-analysis demonstrate that the addition of prehabilitation to standard postoperative rehabilitation significantly reduces PPCs, improves functional capacity, and enhances pulmonary function in patients undergoing cardiac surgery. While effects on length of stay and QoL are less consistent, the overall evidence strongly supports the integration of structured prehabilitation into perioperative care pathways. Future research should prioritize large, multicenter RCTs with standardized interventions and long-term follow-up to establish the durability of these benefits and identify the most effective multimodal approaches.
